# Supplementary figures and images for: Transcriptome Analyses Provide Insights into the Auditory Function in Trachemys scripta elegans
Source: Animals (Basel). 2022 Sep 14;12(18):2410. doi: 10.3390/ani12182410 (PMC9495000; doi:10.3390/ani12182410)

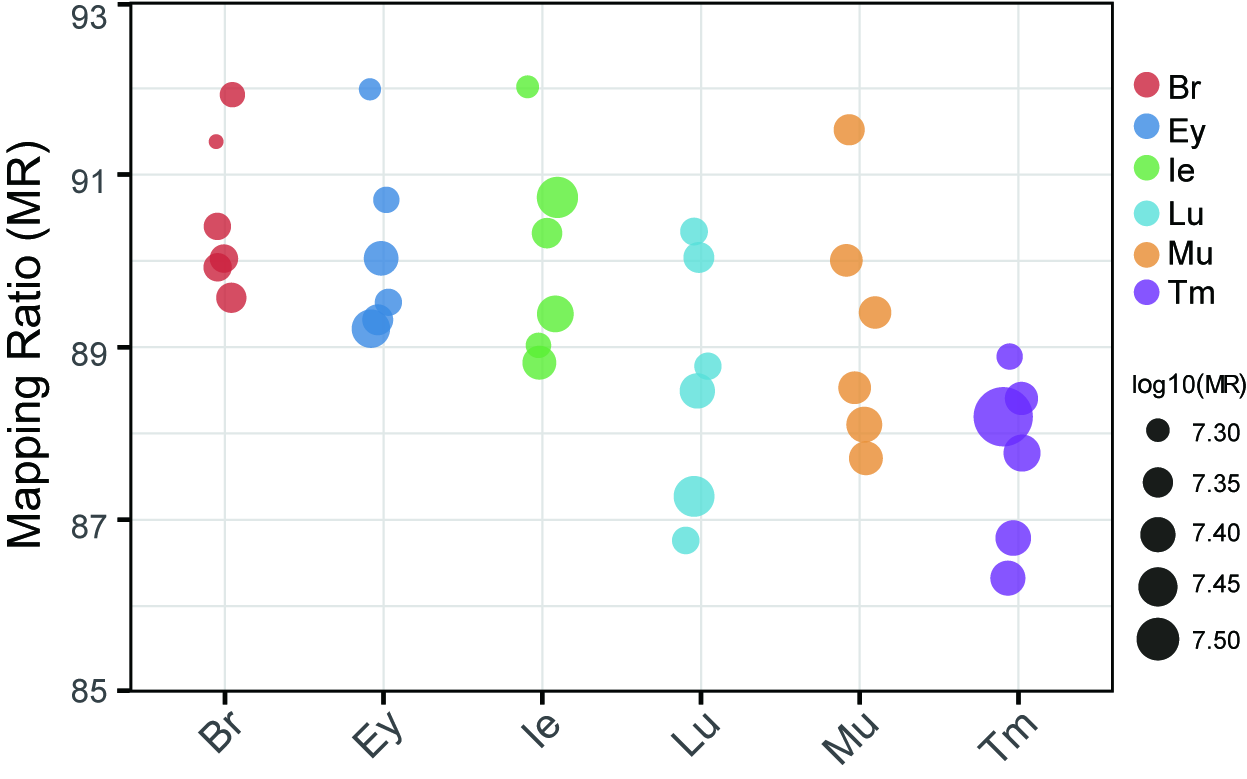

Supplement: Supplementary file 1 [file animals-12-02410-s001.zip › Supplementary Files/Figure S1.tif]

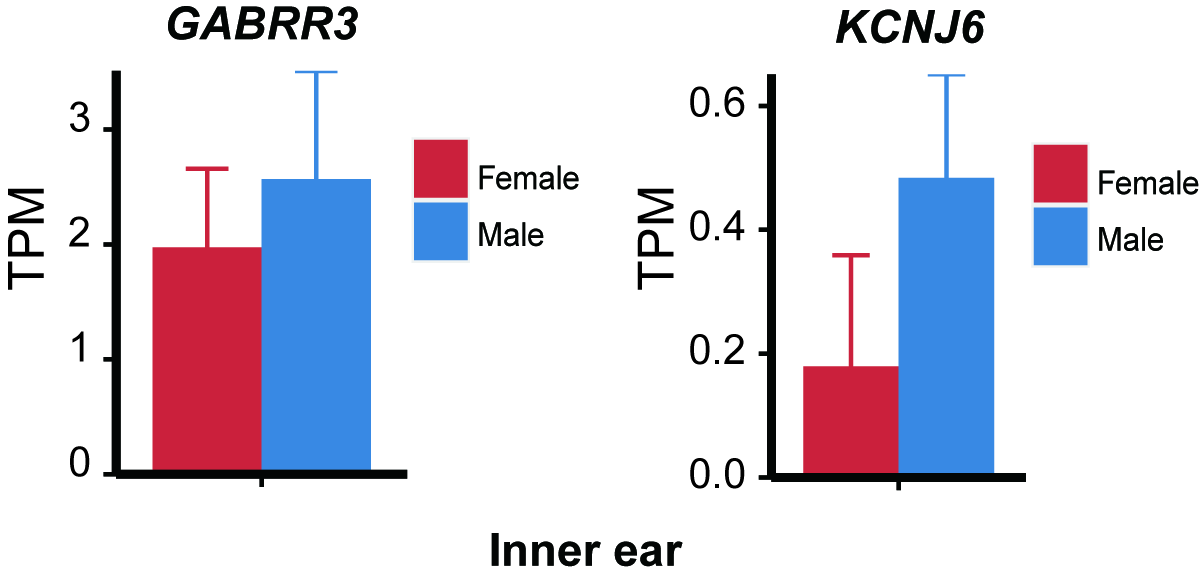

Supplement: Supplementary file 1 [file animals-12-02410-s001.zip › Supplementary Files/Figure S2.tif]
